# Expression of laminin 5-*γ*2 chain in cutaneous squamous cell carcinoma and its role in tumour invasion

**DOI:** 10.1038/bjc.2011.283

**Published:** 2011-08-09

**Authors:** H Hamasaki, K Koga, M Aoki, M Hamasaki, N Koshikawa, M Seiki, H Iwasaki, J Nakayama, K Nabeshima

**Affiliations:** 1 Department of Pathology, Fukuoka University Hospital, 7-45-1 Nanakuma, Jonan-ku, Fukuoka 814-0180, Japan; 2 Department of Dermatology, Fukuoka University Hospital and School of Medicine, 7-45-1 Nanakuma, Jonan-ku, Fukuoka 814-0180, Japan; 3 Division of Cancer Cell Research, Institute of Medical Science, University of Tokyo, Minato-ku, Tokyo 108-8639, Japan

**Keywords:** cutaneous squamous cell carcinoma, laminin 5-*γ*2, tumour invasion, Bowen’s disease

## Abstract

**Background:**

Laminin-5 (Ln5), a heterotrimer composed of three chains (*α*3, *β*3, and *γ*2), is a major component of the basement membrane in most adult tissues. One of the chains, Ln5-*γ*2, is a marker of invasive tumours because it is frequently expressed as a monomer in malignant tumours. Recent studies from our laboratories detected higher levels of Ln5-*γ*2 expression in basal cell carcinoma (BCC) than in trichoblastoma. Furthermore, Ln5-*γ*2 overexpression tended to correlate with aggressiveness in BCC.

**Methods:**

In this study, we compared the expression of Ln5-*γ*2 in invasive squamous cell carcinoma (SCC, *n*=62) of the skin to that in preinvasive Bowen’s disease (BD, *n*=51), followed by analysis of the role of Ln5-*γ*2 in cancer invasion *in vitro*.

**Results:**

Immunohistochemically, the proportion of SCC cases (86%) strongly positive for Ln5-*γ*2 expression was higher than that of BD (16%). Real-time RT–PCR showed Ln5-*γ*2 overexpression in SCC cell line, A431, compared with normal keratinocyte cell line, HaCaT. Ln5-*γ*2 monomer and proteolytically cleaved, biologically active fragments of Ln5-*γ*2 were identified in SCC tumour extracts. In *in vitro* raft cultures, which simulate *in vivo* conditions, Ln5-*γ*2 siRNA significantly suppressed epidermal growth factor (EGF)-stimulated A431 cell invasion.

**Conclusion:**

Our results indicate that Ln5-*γ*2 has a role in cutaneous SCC invasion.

We reported recently more frequent and higher expression of the *γ*2 chain of Ln5 (Ln5-*γ*2) in basal cell carcinoma (BCC) than in trichoblastoma, both of which could arise from the hair follicle epithelium (Hamasaki *et al*, 2011). In addition, Ln5-*γ*2 was expressed more extensively in aggressive subtypes of BCC than in the non-aggressive subtypes. The results suggested the association between Ln5-*γ*2 expression and invading carcinoma cells. These results encouraged us to investigate the expression of Ln5-*γ*2 in the most common malignant tumours of the skin; squamous cell carcinoma (SCC) and Bowen’s disease (SCC *in situ*, BD), with reference to its relationship to tumour invasion. Squamous cell carcinoma and BD are both assumed to arise from the epidermis, but are quite different in their biological behaviour. The majority of SCC is only locally aggressive, but tumours with deep invasion, poor differentiation, perineural invasion, and acantholytic features are more likely to recur or metastasise (Kao *et al*, 2006; Weedon *et al*, 2006). Bowen's disease is a form of SCC *in situ*, and thus a preinvasive lesion with full-thickness squamous cell atypia (Kao *et al*, 2006; Weedon *et al*, 2006).

Laminin-5, a heterotrimer composed of three different laminin chains (*α*3-, *β*3-, and *γ*2-chains), is the major component of the basement membrane in most adult tissues (Nguyen *et al*, 2000). Laminin-5 is an adhesion substrate for epithelial cells, and regulates epithelial cell migration during epithelial regeneration and repair processes (Nguyen *et al*, 2000; Hlubek *et al*, 2001). One of the chains, Ln5-*γ*2, is a specific marker for invasive tumours because it is frequently expressed as a monomer in several types of malignant tumours (Nguyen *et al*, 2000; Koshikawa *et al*, 2008). Several immunohistochemical studies have shown that Ln5-*γ*2 is expressed in tumour cells at the invasion front or in budding tumour cells in many types of human cancers such as adenocarcinomas of the colon, breast, pancreas and lung, SCC of the oesophagus, and melanomas (Pyke *et al*, 1995; Soini *et al*, 1996; Sordat *et al*, 1998; Maatta *et al*, 1999; Hlubek *et al*, 2001; Yamamoto *et al*, 2001; Baba *et al*, 2008).

To our knowledge, there are no comparative studies on Ln5-*γ*2 expression in invasive SCC and preinvasive BD. We report here higher Ln5-*γ*2 expression levels in SCC than in BD. Moreover, active fragments of Ln5-*γ*2 were identified in human SCC tissue extracts. Together with the results of *in vitro* invasion assays using Ln5-*γ*2 small interfering RNA (siRNA), the findings indicate that Ln5-*γ*2 has a role in cutaneous SCC invasion.

## Materials and methods

### Tissue samples

The study material comprised 62 SCC from 29 males and 33 females (age, range, 43–98 (mean, 77) years) and 51 BD from 19 males and 32 females (age, range, 23–99 (mean, 78) years) obtained from the skin tumour files of the Department of Pathology, Fukuoka University Hospital between 1995 and 2011. The study protocol was approved by the Ethics Committee of Fukuoka University.

### Immunohistochemistry

Immunoperoxidase staining of formalin-fixed, paraffin-embedded tissue sections was performed using a standard immunoperoxidase technique (Histofine SAB-PO Kit, Nichirei Biosciences, Tokyo, Japan). Briefly, the tissue sections were deparaffinised, rehydrated in descending alcohol dilutions, followed by treatment with 0.05% Protease XXIV (Sigma-Aldrich, Tokyo, Japan) for 15 min. After blocking nonspecific sites with 5% non-fat dry milk for 10 min, the sections were incubated at 37 °C for 1 h with anti-human Ln5-*γ*2 monoclonal antibody (D4B5, Chemicon, Temecula, CA, USA) at a dilution rate of 1 : 200. The sections were then washed in Tris-buffered saline (TBS) and incubated with Histofine reagent conjugated to alkaline phosphatase (Nichirei Biosciences) for 1 h at room temperature. The reaction was identified with naphthol AS-BI phosphate and New Fuchsin, and counterstained with methylgreen. The stained sections were evaluated semiquantitatively by two independent observers who were blinded to the clinical data. Immunostaining was considered negative if <10% of the tumour cells were stained. In specimens considered positive, staining of the tumour was quantitated on a scale from 1 to 4 based on the percentage of positively stained tumour cells. The scale was structured as follows: 1+=10–25% 2+=25–50% 3+=50–75% and 4+>75%.

### Cell cultures

Human skin SCCs (A431, American Type Culture Collection, Manassas, VA, USA) and HaCaT human keratinocytes (Cell Line Service, Eppelheim, Germany) were cultured in Dulbecco’s modified Eagle’s medium (DMEM), high glucose supplemented with 10% fetal calf serum (FCS), streptomycin (50 *μ*g ml^−1^), and penicillin G (50 U ml^−1^) (growth medium).

### Protein extraction from frozen tissue samples and western blotting

Tumour tissues were obtained from portions of the tumour including the invasion front, whereas control tissues were obtained from normal skin epidermis. All specimens were frozen immediately in liquid nitrogen, embedded in tissue compound, and kept at −80° C until use. Cancer portions and non-neoplastic epidermis portions were dissected manually under stereomicroscope from frozen sections of SCC and normal skin, respectively. The dissected frozen tissues were lysed in RIPA lysis buffer (50 mM Tris-HCl, pH 7.4, 150 mM NaCl, 1 mM EDTA, 1% NP-40; Millipore, Bedford, MA, USA), and the lysed cells were sonicated on ice for 5 min three times, and centrifuged at 15 000 r.p.m. for 20 min at 4 °C. The resultant supernatants were subjected to sodium dodecyl sulfate–polyacrylamide gel electrophoresis (SDS–PAGE). After electrophoresis, the proteins were transferred electrophoretically to Immobilon membrane (Millipore). Nonspecific sites were blocked with 5% dry fat milk in TBS at 37 °C for 1 h and the membrane was incubated overnight at 4 °C with anti-Ln5-*γ*2 antibody and anti-MT1-MMP (membrane type 1-matrix metalloproteinase, AB815, Millipore) antibodies. After washing with TBS-T (TBS containing 0.05% Tween-20), the membrane was incubated for 1 h with peroxidase-conjugated anti-mouse IgG. Colour was developed with chemiluminescence reagents according to the instructions supplied by the manufacturer (DuPont NEN, Boston, MA, USA).

### RNA extraction, cDNA synthesis, and real-time reverse transcription polymerase chain reaction

Total RNA was isolated from frozen tissue specimens, using illustra RNAspin mini RNA isolation kit (GE Healthcare Bio-Sciences, Piscataway, NJ, USA), according to the instructions supplied by the manufacturer. Next, the cDNA was synthesised from 1 *μ*g of total RNA, using Ready-To-Go T-Prime First Strand Kit (GE Healthcare BioSciences). Gene-specific oligonucleotide primer pairs for Ln5-*γ*2 (LAMC2) obtained from Sigma-Aldrich (St Louis, MO, USA) and glyceraldehyde-3-phosphate dehydrogenase (GAPDH) obtained from Takara Bio (Otsu, Japan) were as follows: LAMC2: forward, 5′-GATGGCATTCACTGCGAGAAG-3′ reverse, 5′-TCGAGCACTAAGAGAACCTTTGG-3′ and GAPDH: forward, 5′-GCACCGTCAAGGCTGAGAAC-3′ reverse, 5′-TGGTGAAGACGCCAGTGGA-3′. Primer pairs for ordinary RT–PCR for MT1-MMP and MMP-2 were as follows: MT1-MMP: forward, 5′-TCGGCCCAAAGCAGCAGCTTC-3′ reverse, 5′-CTTCATGGTGTCTGCATCAGC-3′ and MMP-2: forward, 5′-GTGCTGAAGGACACTAAAGAAGA-3′ reverse, 5′-TTGCCATCCTTCCTCAAAGTTGTAGG-3′. Real-time monitoring of PCR reactions was performed using the Light-Cycler system (Roche Applied Science, Indianapolis, IN, USA) and SYBRVR Premix Ex Taq II (Takara Bio), according to the instructions supplied by the manufacturer. Each assay was performed three times to verify the results. The normalised values for cutaneous SCC and corresponding normal skin epidermis were analysed statistically by using the Student’s *t*-test. The PCR products of MT1-MMP and MMP-2 were analysed by electrophoresis on 2% agarose gel stained with ethidium bromide.

### Small interfering RNA

Cells (A431) were grown to preconfluence and treated with siRNA for Ln5-*γ*2 and MT1-MMP (Thermo Fisher Scientific, Waltham, MA, USA) or control siRNA (B-Bridge International, Sunnyvale, CA, USA) using Lipofectamine 2000 (Invitrogen, Carlsbad, CA, USA) according to instructions provided by the manufacturer. The 19-bp targeting sequences were 5′-GGGUGGUGAUGGAGUAGUA-3′ for Ln5-*γ*2 and 5′-GGAUGGACACGGAGAAUUU-3′ for MT1-MMP.

### Detection of Ln5-*γ*2 and MT1-MMP protein in siRNA-treated tumour cells

Cells (A431; 3.0 × 10^4^ cells ml^−1^) were seeded in six-well plates in serum-free DMEM. After stabilisation of the cells over a 24-h period, the medium was aspirated, and replaced with serum-free DMEM, with or without siRNA transfection. After removal of the medium, the cells were washed and scraped in PBS and collected by centrifugation at 1000 r.p.m. for 5 min. The cells were then lysed with RIPA lysis buffer containing protease inhibitor cocktail tablets (Complete Mini, Roche Applied Science, Mannheim, Germany). Lysed cells were sonicated on ice for 5 min, three times, and centrifuged at 15 000 r.p.m. for 20 min at 4 °C. The resultant supernatants were subjected to SDS–PAGE as described above.

### *In vitro* Matrigel invasion assay

The cells were treated with OPTI-MEM containing 0.02 pmol ml^−1^ siRNA for Ln5-*γ*2, MT1-MMP or control siRNA for 48 h. After the treatment, the invasion assay was performed as described previously using modified fluoroblock invasion assay (BD BioCoat Tumour Invasion system, BD Biosciences, Franklin Lakes, NJ, USA) (Aoki *et al*, 2007). Cells (A431) labelled with 10 ng ml^−1^ Di1 (Invitrogen) (3.8 × 10^5^ ml^−1^) were placed in the top compartment of a fluoroblock 24-multiwell insert plate, which was separated from the bottom compartment by a Matrigel (25 mg)-coated fluoroblock membrane with 8.0-*μ*m pore size. Serum-free DMEM with or without epidermal growth factor (EGF) (10 ng ml^−1^) were added into the bottom compartment, and 10% FCS added to the bottom compartment served as a positive control. After incubation for 24–72 h at 37 °C in a 5% CO_2_ atmosphere, the labelled A431 cells, that had invaded through the Matrigel, were scanned by the bottom-reading type fluorescence plate reader (Labsystems Fluoroskan Ascent, Thermo Electron Co., Waltham, MA, USA) at 544/590 nm (absorption/emission). The mean values of the readings and their s.e.m. were calculated, and statistical differences were analysed using Student's *t*-test for non-paired samples.

To determine which MMP was involved in A431 cell invasion, Matrigel invasion experiments were performed in the presence of the following MMP inhibitors: BB-94 (10 or 50 *μ*M; Tocris bioscience, Bristol, UK), TIMP-1 (tissue inhibitors of metalloproteinase-1), and TIMP-2 (10 *μ*g ml^−1^; Fuji Chemical Industries, Takaoka, Japan). The cells were pretreated with inhibitors for 2 h at 37 °C before being added to the upper chamber.

### Raft culture

Human dermal fibroblasts were mixed into a neutralised type I collagen gel (Cellmatrix type I-A, Nitta Gelatin Inc., Osaka, Japan). The mixture was placed into six-well plates (3 ml per well) and allowed to harden for 30 min in a CO_2_ incubator at 37 °C in humidified air. Cells (HaCaT) and A431 cells were then dispensed onto each gel at a density of 1 × 10^6^ cells in 3-ml growth medium. The following day, the hardened gels were detached from the plates, and incubation was continued for 1 week (Ikebe *et al*, 2007). The contracted-gel discs were then placed on the mesh of the cell strainers (BD Biosciences) placed on a fresh six-well plate so that the HaCaT or A431 cells laid on the top of gel discs. The fluid level was adjusted to just below the upper edge of the gel to expose the gel surface to air. Throughout the experiment, one half of the culture fluid was renewed every other day. After 1 week of the air–liquid interface culture, the gel discs were fixed in a 10% phosphate-buffered formalin solution, embedded in paraffin, and vertical sections were stained with hematoxylin and eosin or anti-pancytockeratin antibody (AE1/AE3, Dako, Tokyo, Japan). For raft culture experiments with Ln5-*γ*2 siRNA, the cells were treated with OPTI-MEM containing 0.02 pmol ml^−1^ siRNA for Ln5-*γ*2 or control siRNA every other day (days 0, 2, 4, 6, and 8) (Mitsui *et al*, 2006). After 10 days (4 days of culture within growth medium and 6 days of air–liquid interface culture), the gel discs were fixed and stained as described above. The areas of invading carcinoma cells in each specimen were analysed statistically by using BZ analyser (KEYENCE, Osaka, Japan).

### Statistical analysis

Statistical analysis was conducted using StatView software (SAS Institute, Cary, NC, USA). Differences in clinicopathological parameters between histopathological groups and subgroups were evaluated by *χ*^2^-test, Fisher's exact test or Mann–Whitney *U*-test, while those of *in vitro* studies were evaluated by the Student's *t*-test. A *P*-value of <0.01 and <0.05 was considered statistically significant for the Student's *t*-test and *χ*^2^- and Fisher's exact tests, respectively.

## Results

### Clinicopathological findings

Table 1 summarizes the clinical characteristics of 62 patients with SCC and 51 patients with BD. For SCC, 37% (23 out of 62) of the lesions were located on the face and sun-exposed parts, in agreement with many reports of SCC on the sun-exposed skin, such as the forehead, face, ears, scalp, neck, and dorsum of the hands (Kao *et al*, 2006; Weedon *et al*, 2006). Bowen's disease tended to occur more frequently in females than males, whereas SCC showed no gender predilection.

### Expression of Ln5-*γ*2

**Figure 1:**
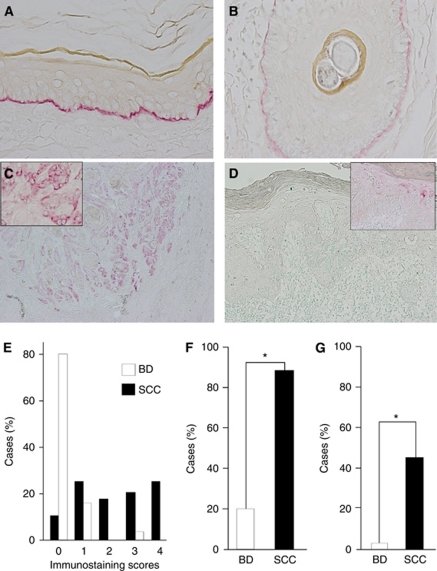
Immunohistochemical analysis of Ln5-*γ*2 expression in non-neoplastic skin, squamous cell carcinoma (SCC), and Bowen's disease (BD). (**A**, **B**) Non-neoplastic skin showed Ln5-*γ*2 immunoreactivity along the basement membrane of the epidermis (**A**) and hair follicle epithelium (**B**). (**C**) Squamous cell carcinoma showed Ln5-*γ*2 immunoreactivity in the cytoplasm of tumour cells, especially in the peripheral portion of tumour nests and the invasion front. (**D**) Bowen's disease showed only a small number of positive cells within the neoplastic epidermis. (**E**) Semiquantitative analysis of Ln5-*γ*2 expression in SCC and BD. (**F**) Comparison of the proportion of cases positive for Ln5-*γ*2 expression between SCC *vs* BD. (**G**) Comparison of the proportion of cases with high expression (scores 3 and 4) between SCC *vs* BD. Insets in (**C**) and (**D**), magnified views of positive tumour cells. Data are mean±s.e.m. ^*^P<0.01 by Fisher's exact test.

In the normal skin, Ln5-*γ*2 was expressed linearly along the basement membrane of the epidermis and hair follicle epithelium (Figure 1A and B). In contrast, SCC cells showed cytoplasmic immunoreactivity to Ln5-*γ*2, although the numbers and distribution of positive tumour cells varied widely in tumours. Positive immunoreactivity tended to be more frequent in SCC than BD. Figure 1C shows a representative case of SCC, in which the Ln5-*γ*2 immunoreactivity was seen both in the periphery of tumour nests and the invasion front. Reactivity of Ln5-*γ*2 was noted in the cytoplasm of tumour cells (Figure 1C inset). However, in cases of BD, the expression of Ln5-*γ*2 was mostly negative (Figure 1D); only a few cases were positive and such immunoreactivity was limited to a small proportion of tumour cells within the epidermis (Figure 1D inset).

Figure 1E–G show a summary of the results. Most cases of SCC (86%, 53 out of 62) were positive for Ln5-*γ*2, with varying staining scores (Figure 1E), whereas only 16% (8 out of 51) of BD cases were positive (Figure 1F, *P*<0.01 by Fisher's exact test). Moreover, 40% (25 out of 62) of the Ln5-*γ*2-positive SCC cases were scored 3+ and 4+ (high-expression group), whereas only 2% (1 out of 51) of the positive BD cases showed high expression of Ln5-*γ*2 (Figure 1G, *P*<0.01 by Fisher's exact test). The high-expression group (score 3+ and 4+) showed more diffuse Ln5-*γ*2 expression within tumours, while the low expression group (score 1+ and 2+) showed focal Ln5-*γ*2 expression at the periphery of tumour nests or only at the invasion front. In addition, in SCC, we examined the correlation between the extents of Ln5-*γ*2 expression and clinicopathological parameters (*n*=62). No statistically significant differences were demonstrated between high- and low-expression groups for the age, sex, differentiation, tumour thickness, tumour grade, and lymph node metastasis (Table 2).

### Detection of Ln5-*γ*2-related proteins in human cutaneous SCC tissues

**Figure 2:**
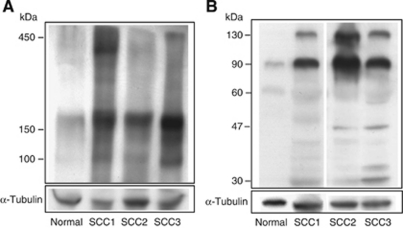
Detection of Ln5-*γ*2 and its fragments in tissue extracts from non-neoplastic epidermis and cutaneous SCC by western blotting. (**A**) Under non-reducing conditions, tissue extracts of the normal epidermis and SCC showed a vague and broad 400–450 kDa band. A 150–180 kDa band was also noted in both tissue extracts, although its intensity was stronger in SCC than normal epidermis. Only SCC tissue showed a band at 100 kDa. (**B**) Under reducing conditions, both normal skin and SCC tissues showed 130- and 90-kDa bands, although their intensities were stronger in SCC than in normal tissues. Smaller 47- and 30-kDa fragments were also generated in SCC tissue extracts. SCC1-3, three different cases of human SCC.

On the basis of the high expression levels of Ln5-*γ*2 in SCC, we also examined the expression of Ln5-*γ*2 protein in SCC tissues using immunoblot analysis. Under non-reducing conditions, tissue extracts from both normal epidermis and SCC showed a blurred and broad band around 400–450 kDa and a smaller 150–180 kDa band (Figure 2A). In comparison, a 100-kDa band was seen in SCC tissue extracts. The intensity of the 150–180 kDa band was also stronger in SCC than in the normal epidermis. Under reducing conditions, high-molecular-weight bands (400–450 kDa) were not detectable in both non-neoplastic epidermal and SCC tissues, but instead 130- and 90-kDa bands were generated (Figure 2B). These two bands of Ln5-*γ*2-related proteins were more common in SCC than in the normal epidermis. Faint and smaller 47- and 30-kDa bands were also generated in SCC tissue extracts.

### Expression of Ln5-*γ*2 mRNA and protein in A431 and HaCaT cell lines

**Figure 3:**
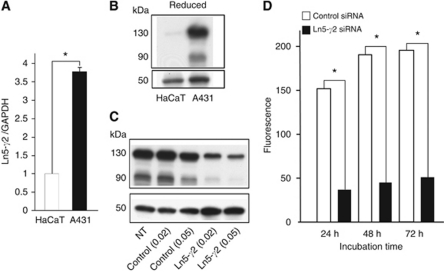
Expression of Ln5-*γ*2 in cultured cell lines and effect of Ln5-*γ*2 siRNA on tumour invasion. (**A**) Real-time RT–PCR expression level of Ln5-*γ*2 mRNA was higher in A431 SCC than in HaCaT non-neoplastic keratinocytes. (**B**) Ln5-*γ*2 protein expression level by western blotting. The intensity of the 130- and 90-kDa bands of the Ln5-*γ*2 chain was stronger in A431 cells than in HaCaT cells. (**C**) Treatment with Ln5-*γ*2 siRNA downregulated the expression of 130- and 90-kDa bands of Ln5-*γ*2. (**D**) Tumour cell invasion of Matrigel was significantly suppressed by transfection of Ln5-*γ*2 siRNA (0.02 pmol ml^−1^) at 24, 48, and 72 h. Data in (**A**) and (**D**) are shown as mean±s.e.m. (*n*=4) ^*^P<0.01 by Student's *t*-test. Control=control siRNA; NT=non-treated.

Next, the expression levels of Ln5-*γ*2 mRNA and protein were examined in cultured cell lines, A431 SCC cells and HaCaT non-neoplastic epidermal cells. The Ln5-*γ*2 mRNA was expressed at significantly higher levels in A431 cells than in HaCaT cells (Figure 3A). Western blotting showed that A431 and HaCaT cells predominantly expressed 130- and 90-kDa forms of Ln5-*γ*2 protein, which corresponded to the full size and the domain I-III of the *γ*2 chain, respectively (Figure 3B). These Ln5-*γ*2 proteins were also expressed at higher levels in A431 cells than HaCaT cells.

### Effect of Ln5-*γ*2 siRNA on invasive ability of SCC cells

#### Matrigel invasion assay

Treatment of A431 cells with Ln5-*γ*2 siRNA (0.02 and 0.05 pmol ml^−1^) downregulated Ln5-*γ*2 expression at 48 (Figure 3C) and 72 h (data not shown) after transfection. To determine the role of Ln5-*γ*2 in carcinoma cell invasion, we first examined the effect of Ln5-*γ*2 siRNA on Matrigel invasion by tumour cells. Epidermal growth factor (10 ng ml^−1^) induced a significant A431 cell invasion, and this EGF-induced invasion was significantly suppressed by transfection of Ln5-*γ*2 siRNA (0.02 pmol ml^−1^) (Figure 3D).

#### Raft culture assay

**Figure 4:**
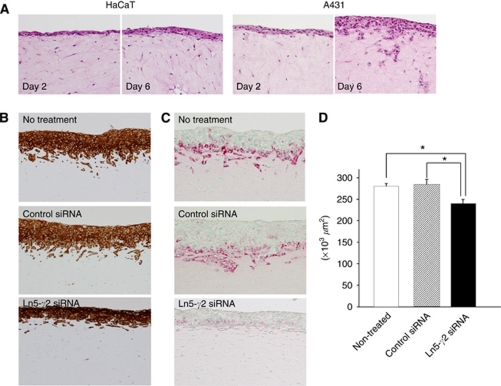
Effect of Ln5-*γ*2 siRNA on tumour cell invasion examined by the raft culture assay. (**A**) Histological findings on days 2 and 6 of raft culture. Human A431 SCC cells invaded the fibroblast-embedded collagen gel, while HaCaT normal keratinocytes did not. (**B**, **C**) Histological findings of invading tumour cells. The invading tumour cell layers were thicker in non-treated or control siRNA-treated cells than Ln5-*γ*2 siRNA-treated ones. Ln5-*γ*2 expression also reduced in the invasion front. (**D**) Tumour invasion analysed at day 6 of raft culture and expressed as area occupied by invading SCC cells. Treatment with Ln5-*γ*2 siRNA significantly reduced the area of the invading tumour cells. Data are shown as mean±s.e.m. of six experiments. (**A**) Hematoxylin and Eosin staining, (**B**) immunohistochemistry with anti-pancytokeratin (AE1/AE3), (**C**) immunohistochemistry with anti-human Ln5-*γ*2 monoclonal antibody.

The effect of Ln5-*γ*2 siRNA on SCC cell invasion was also examined using the raft culture, which better reflects the *in vivo* condition than Matrigel invasion assay. Without treatment, A431 SCC cells invaded the fibroblast-embedded collagen gel, while HaCaT keratinocytes did not (Figure 4A). Incubation with Ln5-*γ*2 siRNA significantly suppressed carcinoma cell invasion, and this suppression was statistically significant when invasive carcinoma areas were estimated (Figure 4B–D). Under control conditions, the carcinoma cells invaded collagen gels in vertical-penetrating cords or showed cluster formation, whereas the cells tended to spread horizontally and exhibit lesser vertical penetration in the presence of siRNA.

### Role of MMPs in SCC cell invasion of Matrigel

#### Expression of MT1-MMP and MMP-2 in A431 and HaCaT cell lines

**Figure 5:**
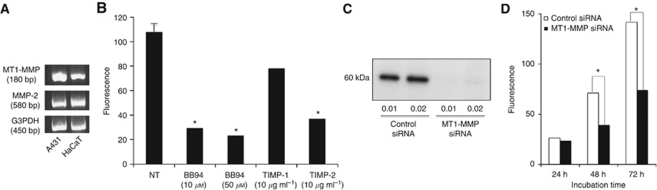
Effect of MMP inhibitors and siRNA on A431 tumour cell invasion. (**A**) RT–PCR detection of MT1-MMP and MMP2 expression in A431 SCC cells and HaCaT non-neoplastic keratinocytes. (**B**) Matrigel invasion assay. BB94 and TIMP-2 significantly blocked A431 tumour cell invasion of Matrigel at 48 h, whereas inhibition by TIMP-1 was insignificant. (**C**) Treatment with MT1-MMP siRNA downregulated the expression of 60 kDa bands of MT1-MMP. (**D**) Tumour cell invasion of Matrigel was significantly suppressed by transfection of MT1-MMP siRNA (0.01 pmol ml^−1^) at 48 and 72 h. Data in (**B**) and (**D**) are shown as mean±s.e.m. (*n*=4). ^*^P<0.01 by Student's *t*-test. G3PDH=glyceraldehydes-3-phosphate dehydrogenase.

Expressions of MT1-MMP and MMP-2, which can cleave Ln5-*γ*2 chain, were detected at the mRNA level by RT–PCR in both A431 and HaCaT cells (Figure 5A).

#### Matrigel invasion assay

To determine which of MT1-MMP and MMP-2 has a more critical role in A431 cell invasion, the effects of MMP inhibitors on Matrigel invasion were examined. BB94 significantly blocked tumour cell invasion of Matrigel at 48 h at both 10 and 50 *μ*M. TIMP-2 also significantly inhibited tumour cell invasion of Matrigel at 48 h, while inhibition by TIMP-1 was in significant (Figure 5b). These results suggested a more critical role of MT1-MMP.

To confirm the role of MT1-MMP in A431 cell invasion, experiments with siRNA for MT1-MMP were performed. Treatment with the MT1-MMP siRNA, which effectively downregulated MT1-MMP expression at 48 h after transfection (Figure 5C), significantly suppressed A431 SCC cell invasion at both 48 and 72 h (Figure 5D).

## Discussion

The major findings of the present study were: (1) more frequent and higher expression levels of Ln5-*γ*2 mRNA in cutaneous invasive SCC than in preinvasive BD: SCC cells diffusely expressed Ln5-*γ*2, while BD cells were rarely immunoreactive to Ln5-*γ*2, (2) identification of Ln5-*γ*2 monomer and proteolytically cleaved, biologically active fragments of Ln5-*γ*2 in tissue extracts from invasive SCC, and (3) suppression of Ln5-*γ*2 using Ln5-*γ*2 siRNA significantly inhibited SCC cell invasion. Considered together, the results suggest that carcinoma cell invasion correlates with Ln5-*γ*2 expression level in cutaneous SCC.

Among the tumour invasion-inducible factors, transforming growth factor (TGF)-*β*1, hepatocyte growth factor (HGF), and Ln5-*γ*2 have been reported to be involved in cutaneous SCC tumorigenesis (Nguyen *et al*, 2000). Transforming growth factor-*β*1 has a dual role in skin carcinogenesis. In mice studies targeting TGF-*β*1 transgene, primarily in the suprabasal/differentiated layers of the epidermis in transgenic, overexpression of TGF-*β*1 inhibits papilloma formation at early stages but promotes tumour aggressiveness via epithelial–mesenchymal transition at later stages of skin carcinogenesis (Weeks *et al*, 2001). Hepatocyte growth factor induces tumour cell scattering and invasion by binding to its receptor c-Met, which is expressed on SCC cells (Knowles *et al*, 2009). The *γ*2 chain of Ln5 also induces tumour cell migration and scattering through mechanisms discussed below (Giannelli *et al*, 1997; Schenk *et al*, 2003; Koshikawa *et al*, 2005, 2008). More interestingly, both TGF-*β*1 and HGF upregulate Ln5-*γ*2 expression. Transforming growth factor-*β*1 and HGF synergistically stimulate the *LAMC2* gene encoding the Ln5-*γ*2 via activation of *cis*-elements including AP-1 (Olsen *et al*, 2003). The transcripts encoding other Ln5 chains are not synergistically activated by TGF-*β*1 and HGF. Thus, this synergistic activation of the LAMC2 gene results in overproduction of Ln5-*γ*2 relative to other Ln5-constituent chains. This difference may explain the accumulation of Ln5-*γ*2 in the cells at the invasive front of tumours. Expression of Ln5-*γ*2 has been studied in keratinocytes and SCC cells *in vitro* (Klimecki *et al*, 1997), but a few reports that included only a small number of cases are available for that in cutaneous SCC *in vivo* (Pyke *et al*, 1994; Kerkela *et al*, 2001; Natarajan *et al*, 2003). The present study of 62 cases of cutaneous SCC confirmed the previously reported pattern of Ln5-*γ*2 expression, expression at the invasive edges or at the interface of the tumour with the surrounding stroma (Pyke *et al*, 1994; Kerkela *et al*, 2001; Natarajan *et al*, 2003). Moreover, Ln5-*γ*2 was frequently expressed in invasive SCC cells, but not in preinvasive BD cells. Although TGF-*β*1 is expressed in SCC as described above, no expression of TGF-*β*1 has been reported in BD (Furue *et al*, 1997). This may explain the rarity of Ln5-*γ*2-expressing cells in BD.

**Figure 6:**
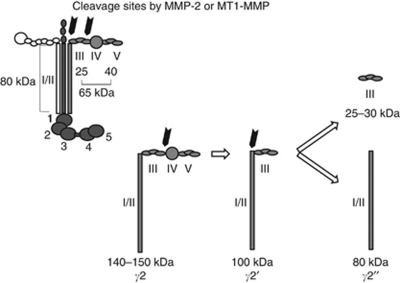
Schematic diagram of the domain structures of the Ln5 heterotrimer and *γ*2 chain. The possible MMP-2 and MT1-MMP cleavage products (*γ*2′, *γ*2″) of *γ*2 chain are also shown. Modified from Koshikawa *et al*, 2000.

The Ln5-*γ*2 chain is a 140-kDa polypeptide and forms a triple helix with the other subunits at its C-terminal (Figure 6) (Nguyen *et al*, 2000), and is frequently expressed as a monomer in several types of cancer cells in association with the lack of simultaneous expression of Ln5-*α*3 and Ln5-*β*3 chains (Koshikawa *et al*, 2008). Expression of monomeric Ln5-*γ*2 is particularly predominant in the budding cells of tumour masses as observed in various cancers, but not in normal tissues (Pyke *et al*, 1995; Sordat *et al*, 1998; Maatta *et al*, 1999; Hlubek *et al*, 2001). These observations emphasise the importance of monomeric Ln5-*γ*2 as a specific marker for invasive carcinoma. In addition, monomeric Ln5-*γ*2 could also have an important role as a modulator of tumour cell behaviour. The *γ*2 chain of Ln5 has an EGF-like repeat in domain III (DIII), and this portion can be clipped out from Ln5-*γ*2 by the action of MMP, such as MMP-2 and MT1-MMP (Giannelli *et al*, 1997; Koshikawa *et al*, 2000, 2005, 2008; Schenk *et al*, 2003). The released DIII fragment can function as a ligand of the EGF receptor and elicits receptor-mediated intracellular signals, which induce migration and scattering of epithelial and tumour cells. In the present study, under non-reducing conditions, the broad band around 400–450 kDa detected in both non-neoplastic and SCC tissue extracts probably corresponds to the heterotrimeric Ln5 (Figure 2A and 6). Under reducing conditions, the 130- and 90-kDa bands possibly correspond to a full-sized monomeric Ln5-*γ*2 chain and the *γ*2′ fragment composed of domains I/II and III, respectively (Figure 2B and 6). The latter could be generated by proteolytic cleavage as described above and further processed by MT1-MMP to release DIII. A small amount of 30-kDa fragment, corresponding to DIII, was also identified in tumour extracts only. These monomeric forms and cleaved fragments of Ln5-*γ*2 were found more frequently in SCC tissue than in non-neoplastic epidermis. Moreover, the cleaved fragment *γ*2′ was identified under non-reducing conditions in tissue extracts only from SCC, but not from the normal epidermis. Collectively, these findings suggest the generation of biologically active DIII-containing fragments of Ln5-*γ*2 within SCC *in vivo*.

As already described above, MT1-MMP and MMP-2 have been reported to proteolytically cleave Ln5-*γ*2 (Giannelli *et al*, 1997; Koshikawa *et al*, 2000, 2005, 2008; Schenk *et al*, 2003). In our study, however, MT1-MMP was more critical for A431 SCC cell invasion than MMP-2, as TIMP-2, but not TIMP-1, significantly inhibited the invasion. Experiments with MT1-MMP siRNA confirmed the role of MT1-MMP in A431 cell invasion. The importance of MT1-MMP in A431 cell invasion may be partly due to cleavage of Ln5-*γ*2, leading to release of the DIII fragment, although MT1-MMP also degrades the Matrigel component directly or through activation of MMP-2.

Treatment of SCC cells with Ln5-*γ*2 siRNA significantly suppressed carcinoma cell invasion as confirmed by both Matrigel invasion and raft culture assays, indicating the role of Ln5-*γ*2 in SCC cell invasion. The raft culture is considered an organotypic culture, that is, a biologically relevant *in vitro* model suitable for studying the molecular mechanism of the underlying dermal–epidermal or dermal–carcinoma interactions (Maas-Szabowski *et al*, 2000). Hence, the results of this assay are more reliable than other methods. In our study, SCC cells invaded the collagen stroma only in the presence of embedded fibroblasts, whereas non-neoplastic keratinocytes did not invade the stroma even in the presence of fibroblasts. These findings suggest the presence of epithelial/carcinoma cell–fibroblast interactions in SCC. In this regard, it is reported that paracrine interactions between carcinoma cells and fibroblasts in SCC can promote cancer invasion: SCC-derived TGF-*β*1 induce transdifferentiation of stromal fibroblasts to myofibroblasts. In turn, myofibroblasts secrete high levels of HGF, which promote SCC invasion (Lewis *et al*, 2004). We speculate that there is another autocrine loop that enhances carcinoma invasion. This autocrine loop is mediated by Ln5-*γ*2, which is produced by carcinoma cells themselves, proteolytically processed into biologically active DIII fragments as already described, and enhance tumour cell invasiveness. Treatment with Ln5-*γ*2 siRNA probably suppressed this autocrine loop. Although the suppression was significant, it was limited to only about 17.5% of the whole invasive area. Inhibition of both autocrine and paracrine loops that promote SCC invasion may be more effective. As HGF in the paracrine loop also stimulates Ln5-*γ*2 expression (Olsen *et al*, 2003), inhibition of the HGF-c–Met pathway may suppress tumour invasion promoted via both paracrine and autocrine loops. This possibility is currently under investigation in our laboratory.

## Figures and Tables

**Table 1:** Characteristics of patients with squamous cell carcinoma and Bowen’s disease

	**SCC**	**BD**	***P*-value**
*n*	62	51	
			
*Age (years)*			0.38^a^
Mean	77	77.8	
Range	43–98	23–99	
			
*Gender*			0.40^b^
Male	29	19	
Female	33	32	
			
*Localization*			
Head	5	—	
Face	23	15	
Arm	4	2	
Hand	7	3	
Body	9	15	
Vulva	4	5	
Leg	5	8	
Foot	5	1	
Unknown	0	2	

Abbreviations: BD=Bowen's disease; SCC=squamous cell carcinoma.

a By Student's *t*-test.

b By *χ*^2^-test.

**Table 2:** Correlation between Ln5-*γ*2 expression levels and clinicopathological parameters of with squamous cell carcinoma

	**Ln5-*γ*2**		
	**High expression (score 3 and 4) (*n*=25)**	**Low expression (score 1 and 2) (*n*=28)**	***P*-value**
*Age (in years)*			>0.9^a^
⩽60	3	3	
>60	22	25	
			
*Sex*			0.4^a^
Male	9	14	
Female	16	14	
			
*Differentiation*			0.8^b^
Well	22	24	
Moderate	3	3	
Poor	0	1	
			
*Thickness (mm)*			0.1^c^
Mean	8.1	6.9	
Range	2–17	1–24	
			
*Grade*			0.3^b^
0	0	0	
I	14	9	
II	6	15	
III	5	4	
IV	0	0	
			
*Lymph node metastasis*			0.1^a^
(+)	6	2	
(−)	2	6	

*n*=53 (positive cases).

a By Fisher's exact test.

b By Mann–Whitney *U*-test.

c By Student's *t*-test.
